# Left ventricular inflow obstruction due to a coronary arteriovenous fistula: a paediatric case report

**DOI:** 10.1186/s12872-021-02190-4

**Published:** 2021-08-11

**Authors:** Ayako Chida-Nagai, Hirokuni Yamazawa, Takao Tsujioka, Kota Taniguchi, Osamu Sasaki, Gaku Izumi, Nobuyasu Kato, Atsuhito Takeda

**Affiliations:** 1 Department of Paediatrics, Hokkaido University Hospital, Kita 14, Nishi 5, Kita-Ku, Sapporo, Hokkaido 060-8648 Japan; 2 Department of Cardiovascular and Thoracic Surgery, Hokkaido University Hospital, Sapporo, Hokkaido 060-8648 Japan

**Keywords:** Congenital heart disease, Coronary atrioventricular fistula, Left ventricular inflow obstruction, Case report

## Abstract

**Background:**

We report a rare case of left ventricular inflow obstruction from a branch of the left circumflex coronary artery to the right atrium caused by a coronary arteriovenous fistula (CAVF) in a young Japanese male child.

**Case presentation:**

The patient was diagnosed with CAVF following a heart murmur shortly after birth. The left-to-right shunt caused right ventricular volume overload and pulmonary congestion. An emergency surgical intervention was performed for the CAVF on day 6 after birth. However, by 5 years of age, his left ventricular inflow obstruction worsened. We found an abnormal blood vessel originating from the proximal part of a branch of the left circumflex coronary artery, circling the outside of the mitral valve annulus along the medial side of the coronary sinus. As the child gets older, the blood inflow into the left ventricle might get restricted further, resulting in left-sided heart failure.

**Conclusion:**

Our findings suggest that even after CAVF closure surgery, it is essential to monitor for complications caused by progressive dilatation of a persistent CAVF.

## Background

A coronary arteriovenous fistula (CAVF) is defined as an abnormal relationship between a coronary artery and any of the cardiac cavities or great vessels. Here, we report the case of a pre-schooler with CAVF and left ventricular inflow obstruction caused by a dilated fistula cavity.

## Case presentation

A male child was born by normal vaginal delivery at a local hospital at 38 weeks of gestation. A heart murmur was heard on auscultation, and echocardiogram showed a type 2 atrial septal defect, pulmonary hypertension, and a CAVF. The electrocardiographic findings also showed hypervolemia in the right chambers of the heart and ST segment depression in V1 to V4. On the third day of life, he was initiated on diuretic therapy to reduce the right ventricular volume overload. On the fourth day of life, his physician discussed his clinical condition with the attending physician in our hospital.

He was referred to our hospital when he was 5 days old. He was breathing and feeding well. A second echocardiography revealed significant hypervolemia in the right chambers of the heart and coronary sinus enlargement. The left ventricular wall from the basal to mid-lateral level showed hypokinesia. Other findings are described in Table 1. It was likely that the hypokinesia was caused by the steal phenomenon. The electrocardiographic findings suggested the same.

**Table 1:** Echocardiographic findings

Valuable		Patient data		
		6 days old	1.5 months old	5 years old
Supramitral valve point velocity, cm/s				
E wave		NA	NA	190
A wave		NA	NA	91
Mitral valve velocity, cm/s				
E wave		115	120	202
A wave		121	98	176
Diameter of mitral valve, mm (Z-score)		10.0 (0.4)	10.0 (0.1)	19.9 (0.7)
Pulmonary vein velocity, cm/s				
A larger mid- and late-systolic component		40	50	56
A diastolic component		51	49	49
LVDd, mm (Z-score)		13.7 (− 2.9)	16.2 (− 1.6)	31.9 (0.4)
LVEF, %		80	79	66

Z-scores are calculated based on Ref. 12

*NA* not measured, *LVDd* left ventricular end-diastolic diameter, *LVEF* left ventricular ejection fraction

**Fig. 1:**
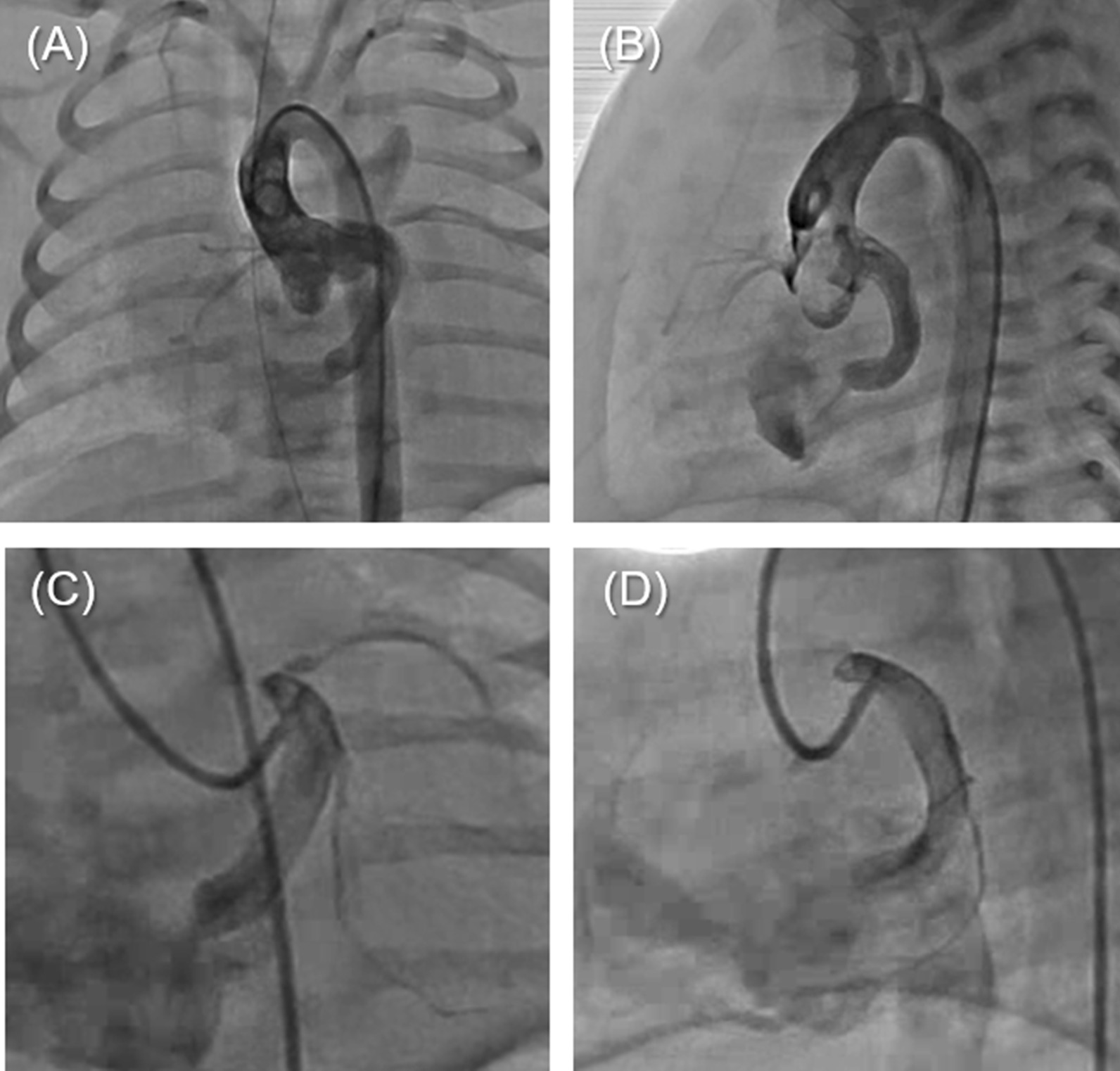
The first cardiac catheterisation shows coronary arteriovenous fistula from a branch of the left circumflex coronary artery to the right atrium. **a**, **c** Frontal image. **b**, **d** Lateral image

Cardiac catheterisation performed on day 6 after birth revealed a CAVF from a branch of the left circumflex coronary artery to the right atrium. From the dilated main trunk of the left coronary artery, the left anterior descending artery followed a normal course. A dilated branch originated from the left main trunk corresponding to the dilated circumflex, which narrowed just before opening into the right atrium, resulting in jet blood flow. The diameter of the abnormal vessel was 6.8 mm (Fig. 1). During cardiac catheterisation, an emergency tracheal intubation had to be performed due to acute respiratory failure; therefore, we could not perform a complete assessment for measurement of the filling pressure and oxygen saturation. Moreover, there was no time to perform a balloon occlusion study for the fistula. An emergency CAVF repair and direct closure of the atrial septal defect were performed on the same day. When a guidewire was inserted into the left circumflex artery, a fistula was identified on the right atrial side, located slightly more cephalad than the coronary sinus. We closed this area with direct sutures. The patient was extubated 2 days postoperatively and discharged 17 days after the surgery. Postoperative echocardiography findings are described in Table 1. Postoperative electrocardiography did not show the ST-T depression, which was seen before the surgery. Postoperative antiplatelet therapy and anticoagulant therapy were administered to prevent coagulation in the coronary arteries. His postoperative course was uneventful; however, echocardiography during regular follow-up showed progressive left ventricular inflow obstruction. At the age of 5 years, he was admitted to our hospital for a detailed examination, although there were no apparent symptoms.

On examination, his height and weight were 102.8 cm (− 1.0 standard deviation [SD]), and 17.0 kg (− 0.3 SD), respectively. The physical examination findings showed a grade 1/6 systolic heart murmur. Serum B-type natriuretic peptide level was 80.2 pg/mL (reference: < 18.4 pg/mL). Chest radiography showed a cardiothoracic ratio of 58% and no abnormal shadows in the lung fields. Electrocardiography showed right atrium enlargement. Echocardiography revealed an abnormal tube-like structure on the mitral valve. Serial flow pattern change (Table 1), and a turbulence flow on colour Doppler suggested left ventricular inflow obstruction.

We simultaneously measured the left pulmonary artery wedge pressure and left ventricular pressure during the second cardiac catheterisation. The mean pressure gradient was 5.8 mmHg. A detailed assessment suggested mild-to-moderate stenosis of the mitral valve. The mean pulmonary artery pressure was 17 mmHg, and the mean pulmonary artery wedge pressure was 11 mmHg.

**Fig. 2:**
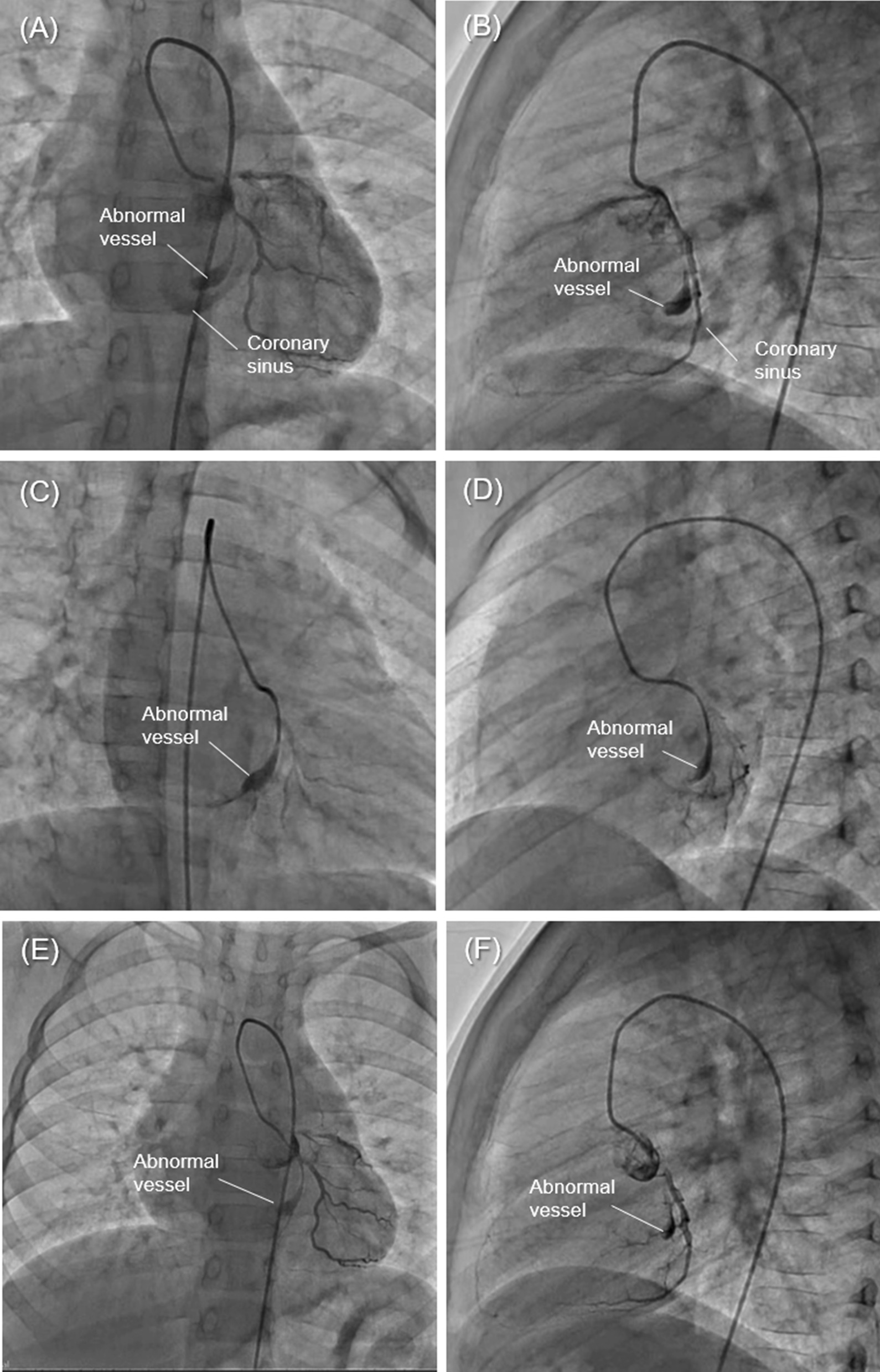
The second cardiac catheterisation shows an abnormal blood vessel originating from the proximal part of a branch of the left circumflex coronary artery branch. **a** Frontal image. **b** Lateral image. **c**–**f** In selective angiography, the vessel’s distal part is blind-ended but thin branches can be seen

An abnormal blood vessel originating from the proximal part of a branch of the left circumflex coronary artery circled the outside of the mitral valve annulus along the medial side of the coronary sinus. The abnormal vessel and the coronary sinus were separate structures (Fig. 2a, b). The diameter at the beginning of this abnormal vessel was 1.6 mm; however, it was extended to 4.5 mm at the distal end. Although the abnormal vessel was interrupted just before the right atrium, a branch from its tip toward the inferior wall of the left ventricle and another branch toward the right atrium were identified (Fig. 2c–f). The pulmonary/systemic blood flow ratio was 1.0.

**Fig. 3:**
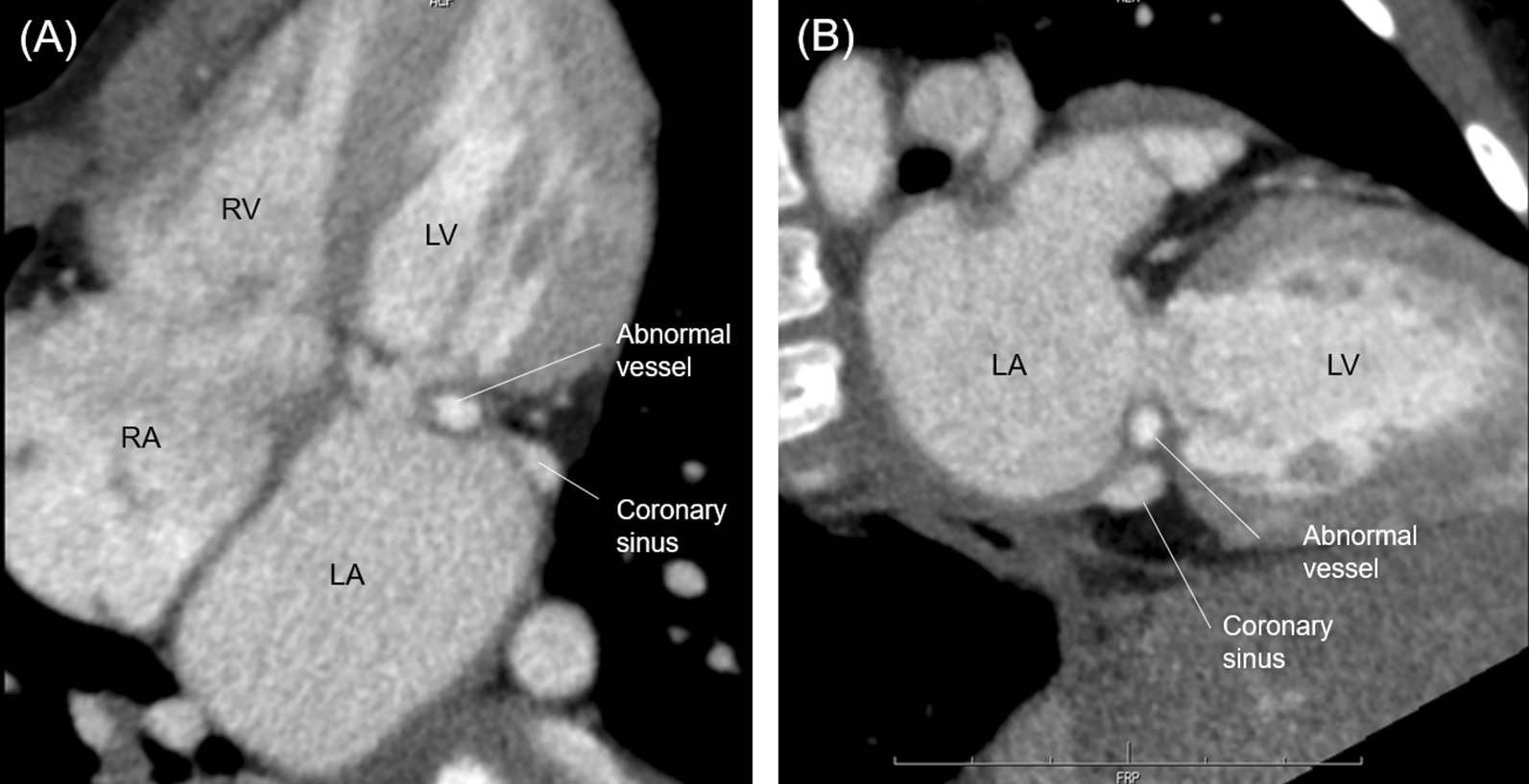
Computed tomography of the patient at 5 years of age revealing left ventricular inflow obstruction caused by an abnormal vessel. **a** Four-chamber image. **b** Two-chamber image

Chest computed tomography revealed a tube-like structure inside the coronary sinus, which could have been a remnant of the CAVF for which he was surgically treated. This abnormal structure relatively narrowed the upper part of the mitral valve (diameter: 11 mm; 69% of the normal mitral valve diameter of 16 mm, the normal range according to body surface area) (Figs. 3a). Based on these findings, we concluded that the remnant CAVF was responsible for the left ventricular inflow obstruction. The echocardiographic evidence of turbulent flow from the site of the abnormal vessel, preserved diameter of the mitral valve annulus, and CT findings suggested that that the main cause of the left ventricular inflow obstruction was the abnormal vessel, not the mitral valve.

We hypothesised that as the child gets older, the blood inflow into the left ventricle would be further restricted and could lead to left-sided heart failure. However, surgical intervention was not indicated at that time as there were no symptoms, pulmonary congestion, or pulmonary hypertension. Two small coronary arteries were found distal to the anomalous vessel; hence, the intervention was abandoned at that time. We decided to monitor the patient on an outpatient basis, and he was continued on warfarin therapy. The patient is currently under careful observation, especially for any findings of ischaemia that can cause arrhythmia or heart failure. To date (the patient is now 7 years of age), he has shown no subjective symptoms, and the degree of left ventricular inflow obstruction has not changed. Based on the course of the disease, it is unlikely that this abnormal blood vessel will suddenly enlarge or rupture.

## Discussion and conclusions

To the best of our knowledge, this is the first case report of left ventricular inflow obstruction in a child caused by persistent CAVF. CAVF is a coronary artery abnormality wherein the coronary arteries open directly into the adjacent cardiovascular space. The incidence of coronary fistulae in the general population is estimated to be 0.002% [1]. More than half of CAVFs originate from the right coronary artery and almost 20% originate from the left circumflex coronary artery [2]. The artery feeding the fistula drains from a coronary artery or one of its branches [3]. In this case, we believe that the fistula originated from an atrial branch from the left circumflex coronary artery. Although the diameter of the abnormal vessel decreased over time, it is likely that as the patient got older, the vessel began to obstruct the left ventricular inflow.

Most CAVFs are small and asymptomatic. However, if left untreated, 19% of the patients are likely to develop clinical symptoms by the age of 20 years and 63% by old age [2]. Treatment is generally indicated for symptomatic patients [4]. Some experts argue that large solitary fistulas causing severe haemodynamic shunting (pulmonary/systemic blood flow ratio ≥ 1.5) should be closed [5]. In this case, we could not measure the pulmonary/systemic blood flow ratio because the patient required emergency tracheal intubation owing to his worsening respiratory status. Therefore, the choice of the first emergency surgical treatment was rational.

The major complications of CAVF are angina, myocardial infarction, pulmonary hypertension, infectious endocarditis, and rupture or thrombosis of the fistula [6], 7. Although several cases of mitral stenosis caused by a fistula have been reported in adult patients with CAVF [8]9, we could not find any paediatric case reports of left ventricular inflow obstruction caused by CAVF.

The best scientifically proven treatment for this condition has yet to be established. After diagnosing his current condition, we opted for enhanced anticoagulation therapy. The indications for warfarin for this patient are controversial; however, we decided to use warfarin with reference to the treatment of large coronary aneurysms as sequelae of Kawasaki disease [10]. If the mitral stenosis aggravates in the future or if the patient develops any symptoms, we plan to first decompress the anomalous vessel by ligation or dissection of the posterolateral branch. If decompression alone is ineffective, suture or dissection of the abnormal vessel from the left atrial side will be performed using an artificial heart lung apparatus. Another treatment option was to ligate the origin of the CAVF and perform coronary artery bypass grafting on the tubular structure. There has been a case report on coronary artery bypass grafting being performed for a similar condition in an adult patient [11]; however, there are no such reports among children. We will continue to monitor the patient's growth and accordingly determine the appropriate timing for surgical intervention.

In conclusion, we described a case of left ventricular inflow obstruction caused by persistent CAVF in a young child. Even after the closure of the CAVF, it is crucial to regularly monitor the patient for complications over the long term in order to facilitate appropriate clinical intervention.

## Data Availability

All data generated or analysed during this study are included in this published article.

## Abbreviation

CAVF: Coronary arteriovenous fistula
